# Landscape of *FGF*/*FGFR* Alterations in 12,372 Chinese Cancer Patients

**DOI:** 10.7150/jca.49269

**Published:** 2020-09-23

**Authors:** Wei Zuo, Yan He, Wei Li, Hao Wu, Zhengyi Zhao, Yuzi Zhang, Shiqing Chen, Yongmei Yin

**Affiliations:** 1 Department of Respiratory, The First Affiliated Hospital of Nanchang University, Jiangxi, China; 2 The Second Shenzhen People's hospital, The First Affiliated Hospital of Shenzhen University, Shenzhen, China; 3 Department of Oncology, The First Affiliated Hospital of Nanjing Medical University, Nanjing, China; 4 The Medical Department, 3D Medicines, Inc. Shanghai, China

**Keywords:** Fibroblast growth factor receptor, Next-generation sequencing, Solid tumor

## Abstract

**Purpose:** The aberrant of fibroblast growth factors and their receptors (*FGF*/*FGFR*) is an emerging target in the treatment of solid tumors. This study aimed to explore the landscape of *FGF*/*FGFR* alterations in a large cohort of cancer patients.

**Material and Methods:** The formalin-fixed paraffin-embedded specimens of cancer patients who have underwent next-generation sequencing (NGS) from 2017 to 2019 in 3DMed Clinical Laboratory Inc. were included in this study.

**Findings:** Of 12,372 Chinese cancer patients with more than 20 tumor types (60% male, median age, 58.0 [IQR, 49.0-66.0]), genomic alterations in FGF, FGFR, and both were observed in 895 (7.2%), 862 (7.0%), and 186 (1.5%) patients, respectively. The highest prevalence of *FGF*/*FGFR* mutations fell in esophagus cancer (61.6%, 98/159) and urinary tract cancer (52.7%, 145/275). The most common pathway-level mutations were *FGFR* single nucleotide variants (635, 5.1%) and *FGF* amplifications (628, 5.1%). The microsatellite instability status was negatively associated with amplifications (*p*=0.0017).

**Conclusion:** *FGF*/*FGFR* alterations were widely occurred in cancer patients, and the mutational landscape may contribute to the further study design and development of *FGF*/*FGFR* inhibitors.

## Introduction

Fibroblast growth factor (FGF) receptors (FGFRs) are highly conserved transmembrane tyrosine kinase receptors (RTKs), which are crucial in a variety of physiological process, such as the regulation of development, differentiation, survival, and migration of cells. Upon the binding of FGF, FGFR kinases will be dimerized and autophosphorylated, thus activating multiple transduction pathways, including the Ras/mitogen-activated protein kinase (MAPK) pathway, the canonical and non-canonical Wnt pathway, and the phosphatidylinositol-4,5-bisphosphate 3-kinase (PI3K)-AKT pathway, which all play importantly roles in the cancer biological process 1-3. The crosstalk between FGF/FGFR with the oncogenic pathways also provide a rationale for the therapeutic strategy for cancer treatment.

In tumor cells, aberrant FGFR signaling can be resulted from the amplification, fusion or missense mutations of FGF or FGFR family members, which are considered as promising targets for cancer treatment 4, 5. In addition, FGF/FGFR signaling has been reported to be mediate resistance to oncotherapy including chemotherapy, radiotherapy and targeted therapy 6, 7. Agents that target FGF/FGFR axis were shown to inhibit angiogenesis and sometimes reverse the acquired resistance of oncotherapy in various cancers 8-10. Currently, more than one hundred clinical trials are ongoing in a variety of advanced cancers with aberrant FGF/FGFR signaling, including several basket trials in solid tumors. Currently, FDA has approved four multikinase inhibitors with FGFR as one of the targets, including ponatinib, regorafenib, pazopanib, and lenvatinib. In April 2019, the first pan-FGFR inhibitor erdafitinib was approved by FDA for the later-line treatment of locally advanced or metastatic urothelial carcinoma with *FGFR*2 or *FGFR*3 genetic alterations based on an ORR of 32.2% from BLC2001 study, a phase 2 trial (NCT02365597) 11, 12. Most recently on April 17, 2020, pemigatinib, an selective inhibitor of *FGFR* isoforms 1, 2 and 3 got approval for *FGFR*2+ cholangiocarcinoma later line treatment upon the data from the FIGHT-202 study with an ORR of 36% 13. In the present study, we aimed to investigate the spectrum of *FGF*/*FGFR* alterations in a large cohort of Chinese cancer patients through next-generation sequencing to aid the future study design and development of *FGF*/*FGFR*-inhibiting drugs.

## Methods

### Clinical specimens

The Formalin-Fixed Paraffin-Embedded (FFPE) specimens of solid tumor patients who have underwent next-generation sequencing (NGS) from 2017 to 2019 in 3DMed Clinical Laboratory Inc. were included. The diagnosis of the specimens was confirmed by hematoxylin and eosin (H&E) staining by an independent pathologist. The specimens were required to have a percentage of tumor cells over 20% and a size ≥1mm for further analysis.

### NGS sequencing

DNAs extracted from the FFPE tumor specimens were sequenced in NGS platform with a well-designed 381 cancer gene panel on Illumina Nextseq 500 to > 500X coverage as previously described^14^ in 3DMed Clinical Laboratory Inc., a College of American Pathologists (CAP) certified and Clinical Laboratory Improvement Amendments (CLIA) certified laboratory of 3D Medicines Inc. Genetic alterations were identified, microsatellite instability status (MSI) was assessed, and clinical information including age, gender, and tumor histology were collected. Germline variants were identified by comparing patient's tumor to the matching blood controls. The detected genetic alteration types included single-nucleotide variants (SNVs), insertions/deletions (indels), copy number variations, and gene rearrangements. *FGF* genes included *FGF3*, *FGF4*, *FGF6*, *FGF10*, *FGF14*, *FGF19*, and *FGF23*. FGFR genes included *FGFR1*, *FGFR2*, *FGFR3*, and *FGFR4*. Gene alterations of other cancer signaling pathways, including RTK/RAS pathway, PI3K pathway, and cell cycle pathway, were also identified^15^.

### Statistical analysis

Data were analyzed with GraphPad Prism (version 7.01, GraphPad Software, USA) and R (version 4.3.1, R Development Core Team). All reported *P* values were two-sided, and *P* < 0.05 was considered statistically significant unless otherwise specified.

## Results

### Patient Characteristics

A total of 12,372 Chinese cancer patients with more than 20 tumor types were included in the study, including 3,557 (29%) lung cancer, 1,433 (12%) liver cancer, 1,310 (11%) colorectal cancer (CRC), 960 (8%) biliary tract cancer, and 758 (6%) and gastric cancer (GC), etc. (**Table 1**). The median age was 58 (IQR, 49-66) and 7,440 (60%) were male patients. NGS was conducted with the FFPE tumor specimens of the patients through surgical resection (n=7,959, 64.3%), biopsy (n=3,734, 30.2%), or other approaches (n=681, 5.5%).

### FGF/FGFR Alterations

**Figure 1:**
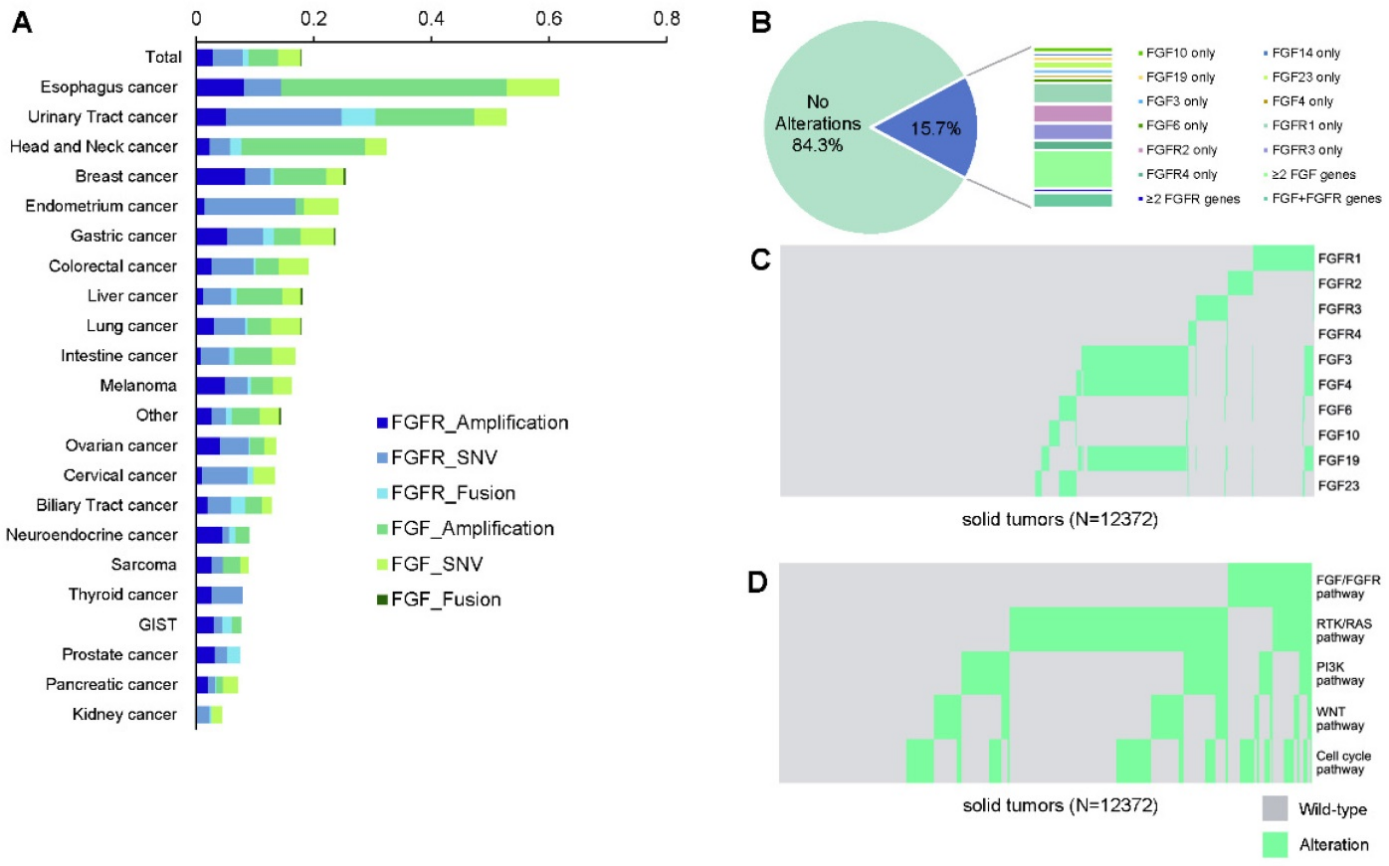
***FGF*/*FGFR* genes in cancer patients.** (A) Landscape of *FGF*/*FGFR* alterations across different cancer types. (B) Distribution of the mutated genes in *FGF*/*FGFR*. (C) The mutual exclusivity and co-occurrence relationship with the corresponding significances (D) among the pathway gene alterations.

Of all patients, FGF/FGFR alterations were observed in 1,943 (15.7%) patients, including 895 (7.2%) with alterations in FGF, 862 (7.0%) in FGFR, and 186 (1.5%) in both FGF and FGFR (**Figure 1A**). Amongst all, there are 1,032 (8.3%) patients with SNVs, 930 (7.5%) with amplifications, and 116 (0.9%) with fusions in FGF/FGFR genes. The alteration frequencies between FGF and FGFR were mostly comparable in each tumor type. FGF/FGFR mutations were widely occurred across all involving tumor types, with a prevalence ranging from 61.6% (98/159) in esophagus cancer, 52.7% (145/275) in urinary tract cancer, 32.3% (83/257) in head and neck carcinoma to 7.4% (7/95) in prostate cancer, 7.1% (45/635) in pancreatic cancer, and 4.4% (24/548) in kidney cancer (**Figure 1A, Table 2**). When stratified by the mutational types, the most common pathway-level abnormalities fell in FGFR genes SNVs (635, 5.1%), FGF genes amplifications (628, 5.1%), and FGFR genes amplifications (357, 2.9%). The top frequent gene-level alterations were amplifications in *FGF19* (462, 3.7%), *FGF4* (454, 3.7%), *FGF3* (438, 3.5%), and *FGFR1* (220, 1.8%), followed by SNVs in *FGFR2* (222, 1.8%), *FGFR3* (211, 1.7%), *FGFR4* (154, 1.2%) and *FGFR1* (146, 1.2%). The somatic mutation maps of *FGFR1/2/3/4* were shown in **Figure 1B**.

### Mutual Exclusivity and Co-occurrence

The relationships between genetic alterations were investigated among FGF/FGFR genes. In brief, alterations in *FGF3*, *FGF4*, and *FGF19* from 11q13 were mostly concomitant, and were partially co-occurred with *FGFR1* alterations (**Figure 1C**). On the other side, most of the other FGF/FGFR genes were mutually exclusive, for example, the alterations of *FGFR1*, *FGFR2*, and *FGFR3* (p<0.001). The mutual exclusivity and co-occurrence mutational pattern was mostly consistent when constrained to gene amplifications, with the co-occurring *FGF3*, *FGF4*, and *FGF19* amplifications, and the widely observed mutual exclusivity across the other genes (data not shown). As the top frequent alterations in the overall population, amplifications in *FGF3/4/19* (490, 4.0%) occurred the most in esophagus cancer (60/159, 37.7%), head and neck carcinoma (54/257, 21.0%), and urinary tract cancer (41/275, 14.9%), and were the least in pancreatic cancer (4/635, 0.6%), sarcoma (2/369, 0.5%), and ovarian cancer (1/293, 0.3%).

In addition, the occurrence of alterations in different pathways were investigated (**Figure 1D**). Significantly higher mutational rates of PI3K pathway (32.0% vs 20.5%, *p*<0.0001), WNT pathway (21.4% vs 18.1%, *p*=0.0010), and cell cycle pathway (49.8% vs 21.9%, *p*<0.0001) were observed in patients harboring FGF/FGFR alterations compared to the wild-type patients, while such trend was not found in RTK/RAS pathway (46.8% vs 48.7, *p*=0.1447).

### Pathway Alterations and Microsatellite Instability

The status of microsatellite instability (MSI) was available in 10,680 (86.3%) of the patients. In overall, 2.4% (258/10,680) patients showed a high MSI (MSI-H) status. Amongst all, MSI-H was found to be positively associated with the FGF/FGFR alterations (p<0.0001), with 124 (48.1%) of the MSI-H patients harbored at least one genetic alteration in FGF/FGFR. However, MSI-H was negatively associated with FGF/FGFR amplifications (p=0.0017), with only seven (2.7%) patients with MSI-H harbored FGF/FGFR gene amplifications.

## Discussion

This is the first study that comprehensively depicting the landscape of FGF/FGFR aberrations in Chinese cancer patients. Our study showed that FGF/FGFR alterations were common across various histologies in Chinese cancer patients.

The recurring oncogenic FGF/FGFR mutations were detected in 15.7% cancer patients, including 7.2% with alterations in FGF genes, 7.0% in FGFR genes, and 1.5% in both FGF and FGFR genes, which is comparable to the previously reported 7% of FGFR alterations in the Caucasian cancer population 16. The distribution of FGFR and FGF alterations were mostly comparable in each of the 20 tumors in the studied cohort, except the dominant FGF alterations observed in esophagus cancer and the dominant FGFR alterations in gynecologic cancer and prostate cancer. For FGFR signaling, the most affected cancer types in the Caucasian cancer population was reported in urothelial (32%), breast (18%), and endometrial and squamous cell lung (13% for each) 16, similar to those in the studied cohort as 30.5% in urothelial cancer, 16.9% in endometrium cancer, and 13.2% in breast cancer. However, the aberrant FGFR signaling was much more common in gastric cancer (19.4% vs 6.7%) and head and neck carcinoma (15.7% vs 4.6%) in Chinese versus Caucasian patients. With the striking results from the drugs targeting mutant *FGF*/*FGFR* in various cancer, non-selective FGF/FGFR inhibitors have been approved for cancer treatment, especially for tumors harboring aberrant *FGFR*2 or *FGFR*3 activations 11, 13. Despite the recently reported disadvantages of a selective inhibitor of *FGFR*1, 2, and 3 in 22 cholangiocarcinoma patients with *FGF*/*FGFR* genetic alterations other than *FGFR*2 translocation from the interim results of flight-202 study 17, the encouraging efficacy of pan-FGFR inhibitor were reported in patients harboring these alterations with durable response 9. In addition, the acquired resistance upon FGF/FGFR mutation for various conventional anti-tumor treatment largely impeded the continued treatment. Of note, amplifications in the 11q13 chromosome (including *FGF*3, *FGF*4, and *FGF*19), which was found to be negatively correlated with MSI-H in this study, was reported to be potentially associated with hyperprogression upon immune checkpoint inhibitors as observed in three out of four patients with lung cancer or esophageal adenocarcinoma 18. Given the different agent sensitivities and efficacies towards various FGF/FGFR genetic alterations, the optimal development of FGF/FGFR axis-target agents required knowledge of the distribution and types of FGF/FGFR aberrations in a variety of cancer types.

There are several limitations in this study. Firstly, given the cross-sectional setting of this study, no treatment or survival data were available for the studied subset, and thus the association between FGF/FGFR alterations with clinical outcome cannot be assessed. Secondly, the cancer subtype was not further identified in the current study, which may lead to potential bias. Collectively, our study has provided valuable clues to the future clinical development, and further investigations are warranted for the clinical application of cancer treatment upon molecular profiling.

## Figures and Tables

**Table 1:** Clinicopathologic Features of 12,372 Chinese Cancer Cases.

Characteristics	All patients (n = 12,372)
Age, median (IQR)	58.0 (49.0-66.0)
Sex, n (%)	
Male	7440 (60)
Female	4932 (40)
Histology type, n (%)	
Lung cancer	3557 (29)
Liver cancer	1433 (12)
Colorectal cancer	1310 (11)
Biliary Tract cancer	960 (8)
Gastric cancer	824 (7)
Pancreas cancer	635 (5)
Kidney cancer	548 (4)
Other	544 (4)
Breast cancer	371 (3)
Sarcoma	369 (3)
Ovarian cancer	293 (2)
Urinary Tract cancer	275 (2)
Head and Neck carcinoma	295 (2)
Cervical cancer	195 (2)
Melanoma	160 (1)
Esophagus cancer	159 (1)
Endometrium cancer	136 (1)
Intestine cancer	124 (1)
Prostate cancer	95 (1)
Neuroendocrine cancer	89 (1)

**Table 2:** Distribution of patients with *FGF*/*FGFR* alterations across different tumor types.

	FGFR Amplification	FGFR SNV	FGFR Fusion	FGF Amplification	FGF SNV	FGF Fusion
**Total**	2.9%	5.1%	0.9%	5.1%	3.9%	0.1%
**Esophagus cancer**	8.2%	6.3%	0.0%	38.4%	8.8%	0.0%
**Urinary Tract cancer**	5.1%	19.6%	5.8%	16.7%	5.5%	0.0%
**Head and Neck cancer**	2.3%	3.5%	1.9%	21.0%	3.5%	0.0%
**Breast cancer**	8.4%	4.3%	0.5%	8.9%	3.0%	0.3%
**Endometrium cancer**	1.5%	15.4%	0.0%	1.5%	5.9%	0.0%
**Gastric cancer**	5.3%	6.2%	1.8%	4.5%	5.7%	0.1%
**Colorectal cancer**	2.6%	7.2%	0.4%	3.9%	5.1%	0.0%
**Liver cancer**	1.2%	4.8%	1.0%	7.7%	3.2%	0.2%
**Lung cancer**	3.1%	5.3%	0.4%	4.0%	5.1%	0.0%
**Intestine cancer**	0.8%	4.8%	0.8%	6.5%	4.0%	0.0%
**Melanoma**	5.0%	3.8%	0.6%	3.8%	3.1%	0.0%
**Other**	2.8%	2.4%	0.9%	4.8%	3.3%	0.2%
**Ovarian cancer**	4.1%	4.8%	0.3%	2.4%	2.0%	0.0%
**Cervical cancer**	1.0%	7.7%	1.0%	0.0%	3.6%	0.0%
**Biliary Tract cancer**	2.0%	4.1%	2.4%	2.8%	1.6%	0.0%
**Neuroendocrine cancer**	4.5%	1.1%	1.1%	2.2%	0.0%	0.0%
**Sarcoma**	2.7%	1.9%	0.0%	3.0%	1.4%	0.0%
**Thyroid cancer**	2.6%	5.3%	0.0%	0.0%	0.0%	0.0%
**GIST**	3.0%	1.5%	1.5%	1.5%	0.0%	0.0%
**Prostate cancer**	3.2%	2.1%	2.1%	0.0%	0.0%	0.0%
**Pancreatic cancer**	2.0%	1.3%	0.2%	1.1%	2.5%	0.0%
**Kidney cancer**	0.2%	2.2%	0.4%	0.0%	1.6%	0.0%
